# Infant Arterial Stiffness and Maternal Iron Status in Pregnancy: A UK Birth Cohort (Baby VIP Study)

**DOI:** 10.1159/000377618

**Published:** 2015-03-12

**Authors:** Nisreen A. Alwan, Janet E. Cade, Harry J. McArdle, Darren C. Greenwood, Helen E. Hayes, Etienne Ciantar, Nigel A.B. Simpson

**Affiliations:** ^a^Nutritional Epidemiology Group, School of Food Science and Nutrition, Leeds, UK; ^b^Division of Biostatistics, University of Leeds, Leeds, UK; ^c^Department of Women's and Children's Health, University of Leeds, Leeds, UK; ^d^Rowett Institute of Nutrition and Health, University of Aberdeen, Aberdeen, UK

**Keywords:** Vascular stiffness, Iron, Pregnancy, Infant, Micronutrients, Anaemia

## Abstract

**Background:**

In animal studies, iron deficiency during pregnancy has been linked to increased offspring cardiovascular risk. No previous population studies have measured arterial stiffness early in life to examine its association with maternal iron status.

**Objective:**

This study aimed to examine the association between maternal iron status in early pregnancy with infant brachio-femoral pulse wave velocity (PWV).

**Methods:**

The Baby VIP (Baby's Vascular Health and Iron in Pregnancy) study is a UK-based birth cohort which recruited 362 women after delivery from the Leeds Teaching Hospitals postnatal wards. Ferritin and transferrin receptor levels were measured in maternal serum samples previously obtained in the first trimester. Infant brachio-femoral PWV was measured during a home visit at 2–6 weeks.

**Results:**

Iron depletion (ferritin <15 µg/l) was detected in 79 (23%) women in early pregnancy. Infant PWV (mean = 6.7 m/s, SD = 1.3, n = 284) was neither associated with maternal ferritin (adjusted change per 10 µg/l = 0.02, 95% CI: −0.01, 0.1), nor with iron depletion (adjusted change = −0.2, 95% CI: −0.6, 0.2). No evidence of association was observed between maternal serum transferrin receptor level and its ratio to ferritin with infant PWV. Maternal anaemia (<11 g/dl) at <20 weeks’ gestation was associated with a 1.0-m/s increase in infant PWV (adjusted 95% CI: 0.1, 1.9).

**Conclusion:**

This is the largest study to date which has assessed peripheral PWV as a measure of arterial stiffness in infants. There was no evidence of an association between markers of maternal iron status early in pregnancy and infant PWV.

## Introduction

Arterial stiffness in adults has been shown to be an independent predictor of cardiovascular events [1], It has also been linked with childhood indicators of cardiovascular risk [2, 3]. Increased arterial stiffness leads to faster return of the reflected pulse wave from peripheral sites to the left ventricle, and hence suboptimal ventricular-arterial interaction [4], Measurement of pulse wave velocity (PWV) is accepted as the most simple, convenient and reproducible method to determine arterial stiffness over the life course [1]. PWV is determined by dividing the distance of the pulse travel between two sites by the transit time, and is inversely related to arterial distensibility [4].

Iron deficiency (ID) is the leading single nutrient deficiency in the world. Maternal ID anaemia has been linked to risk of low birth weight, preterm delivery and infant ID anaemia [5]. There is experimental evidence supporting the involvement of maternal ID in the programming of adult hypertension and obesity. In pregnant rats, ID results in increased risk of offspring obesity and hypertension [6]. Postulated mechanisms include increased placental cytokine and tumour necrosis factor levels, and altered metabolism of mediators of cell function and metals such as copper [7]. ID can also induce changes in placental structure including decreased capillary length and surface area and increased placental vascularisation, and may interfere with fetal kidney development and nephron number [8].

Few studies have examined the relationship between maternal nutritional exposures and childhood arterial stiffness [9, 10, 11]. To our knowledge, there are no population studies assessing such relationships with neonatal or infant arterial stiffness. However, it has been shown that PWV measurements can be reliably ascertained in newborns with good reproducibility [12, 13].

The aim of this study was to investigate the relationship between maternal iron status early in pregnancy with offspring arterial stiffness at 2–6 weeks of life.

## Materials and Methods

### Study Design and Population

**Fig. 1:**
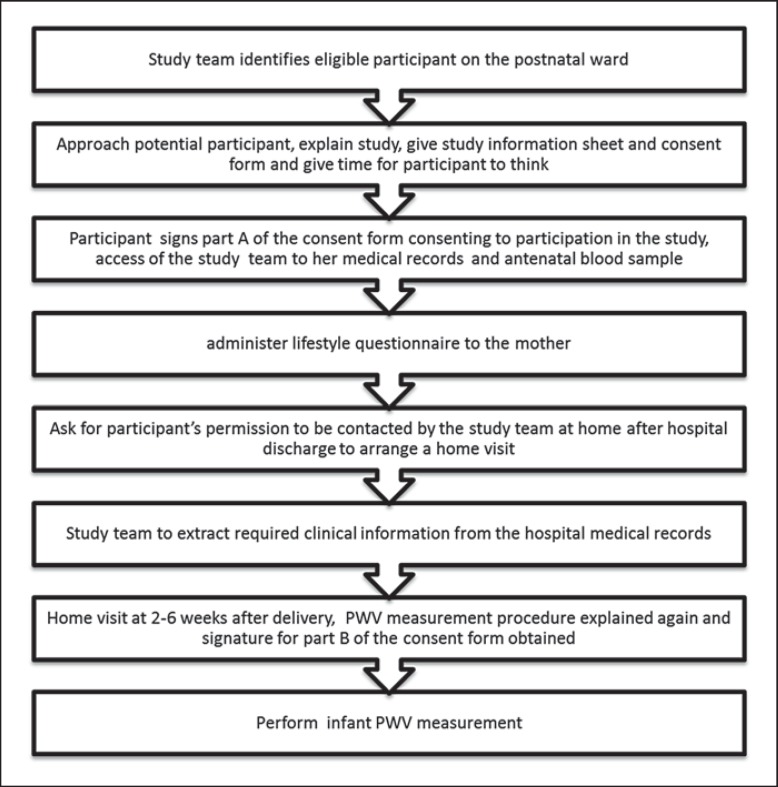
Participant flowchart.

The Baby VIP (Baby's Vascular Health and Iron in Pregnancy) study is a birth cohort study. It comprises women aged ≥18 years who gave birth at the Leeds Teaching Hospitals Trust Maternity Unit at a gestational age of ≥34 weeks between February 2012 and January 2013. The participants were recruited from the postnatal wards after delivery. Those who agreed to take part were asked if the research team could contact them after they were discharged home to arrange a home visit within 6 weeks. Figure 1 illustrates the participant flowchart. Ethical approval was obtained from the South Yorkshire Committee of the NHS National Research Ethics Service (11/YH/0064). All procedures were in accordance with the 1975 Helsinki Declaration as revised in 1983.

### Outcome Measurement

Brachio-femoral PWV (bfPWV) was measured using the Vicorder device (Skidmore Medical), which uses an oscillometric technique. This kit provides a non-invasive method of measuring PWV, and is thought to be relatively independent of operator skills [14]. Two infant-size cuffs were used. The arm cuff was wrapped around the bare skin of the baby's arm, with the mid cuff point halfway between the shoulder and the elbow. The leg cuff was wrapped around the bare skin of the baby's ipsilateral thigh with the mid cuff point halfway between the groin and the knee. Using a tape measure, the distance was measured in centimetres between the two marked points in a straight line while keeping the baby's thigh straight, with the tape kept on the internal side of the arm alongside the trunk. The pressure applied was 35 mm Hg for both the arm and the leg. The pulse recording at the two arterial sites was obtained simultaneously. Transit time was measured as the time delay between the feet of the proximal and the distal pulse waves. A minimum of two PWV readings was obtained from each baby. If they were more than 0.3 m/s different, a third reading was obtained. The average of all available readings for each baby was used in the analyses.

### Exposure Measurement

Serum ferritin (sF) is the most widely used biomarker in the assessment of iron status. The ratio of serum transferrin receptor (sTfR) to sF (R/F) is considered the gold standard marker of iron status [15], and has been used to assess iron status in pregnant populations [16]. Maternal serum samples of 348 women, previously stored during the first trimester of pregnancy as part of routine antenatal care, were analysed. sF was measured using ELISA (Demeditic, Kiel, Germany). 10 µl of plasma were treated with a sandwich ELISA method, using fluorometric measurements and calibrated using standards supplied by the manufacturer. Quality controls were included as appropriate. The WHO cutoff of 15 µg/l in sF was used to indicate depleted iron stores [17].

sTfR assays were performed using a commercially available kit based on a polyclonal antibody in a sandwich enzyme immunoassay format (DTFR1; R&D Systems, Minneapolis, Minn., USA). This yielded sTfR levels in nmol/l units. The values were converted to µg/l using a molecular weight of sTfR of 75,000 Da (R&D technical data sheet). The R/F ratio was obtained by dividing sTfR over sF (µg/l:µg/l). This was logged to obtain normal distribution.

Maternal haemoglobin (Hb) values were extracted from the antenatal care records and/or the hospital electronic results server. A cutoff of 11 g/dl in Hb was used to indicate anaemia at ≤20 weeks’ gestation, and 10.5 g/dl to indicate anaemia beyond 20 weeks’ gestation, following the UK National Institute for Health and Care Excellence guidelines [18].

### Covariable Assessment

The Index of Multiple Deprivation (IMD) was derived using the GeoConvert tool utilising the UK census data (geoconvert.mimas.ac.uk). Birth weight, gestational age, parity, maternal height, weight, ethnicity, smoking, pregnancy complications (pre-eclampsia, gestational diabetes), blood pressure measurements and intake of iron supplements were extracted from the clinical records. Infant heart rate was recorded by the Vicorder device with each PWV measurement.

### Statistical Methods

Statistical analysis was performed using Stata version 11 (2009; College Station, Tex., USA). Customised birth weight centile was calculated taking into account gestational age, maternal height, maternal pre-pregnancy or booking weight, ethnicity, parity, and neonatal sex [19]. Univariable analysis was performed using an independent sample t test, one-way analysis of variance or Mann-Whitney test for continuous variables, and χ^2^ test for categorical variables.

Multiple linear regression was performed with PWV as the main outcome, and indicators of maternal iron status as predictors. The models were adjusted for baby covariables at birth (customised birth weight centile) and at measurement (age, position, measurement side, arousal state and type of feeding), and maternal covariables including age, smoking status, the presence of gestational diabetes or pre-eclampsia, blood pressure at booking and at 36 weeks’ gestation, and IMD deprivation score. Sensitivity analyses were performed taking into account the intake of iron supplements during pregnancy and infant heart rate at the time of PWV measurements.

### Sample Size Calculation

For a difference of 0.3 m/s in PWV between iron-deficient and non-iron-deficient mothers, using a mean of 4.7 m/s, a standard deviation (SD) of 0.6 m/s and a prevalence of ID of 20%, a sample size of 265 mother-baby pairs was required to achieve 90% power with p = 0.05 [12],

## Results

We recruited 362 mother-baby pairs, with 284 (79%) babies going on to have PWV measurements at home. Mean infant bfPWV was 6.7 m/s (SD = 1.3). The mean difference in PWV between the first and the second measurement was −0.02 m/s (95% CI: −0.16, 0.11, Bland-Altman limits of agreements: −2.3 to 2.2 m/s). The with-in-subject coefficient of variation was 1.3% and the intraclass correlation coefficient was 0.6 (95% CI: 0.5–0.7). The difference between the first and second PWV measurement was 0.3 m/s or less in 31% and 0.5 m/s or less in 49% of the babies. Mean baby age at the time of measurement was 25 days (SD = 6). Table 1 describes infant PWV in relation to measurement conditions and baby characteristics. The baby being asleep was, on average, associated with a 0.9-m/s reduction in infant bfPWV (95% CI: 0.5, 1.3). Table 2 describes the characteristics of participants with or without PWV measurements.

Median sF was 31.7 µg/l [interquartile range (IQR): 16.9–62.4]. 79 women (23%) women had depleted iron stores. The median sTfR was 12.8 nmol/l (IQR: 10.2–16.1). Mean maternal Hb was 12.6 g/dl and 11.6 g/dl (SD = 1.0) in the first and second halves of pregnancy, respectively. The prevalence of anaemia at ≥20 weeks’ (<11 g/dl) and >20 weeks’ gestation (<10.5 g/dl) was 5% (16/329) and 14% (48/337), respectively. Only half of the anaemic women in the first half (n = 8) and 45% of the anaemic women in the second half of pregnancy (n = 22) had a first trimester sF of less than 15 µg/l. 121 women (34%) took iron supplements during pregnancy: 8 (2%) started in the first trimester compared to 67 (19%) in the second and 46 (13%) in the third trimester.

There was no evidence of association between infant bfPWV and maternal sF (adjusted change in PWV in m/s per 10-µg/l change in sF = 0.02, 95% CI: −0.01, 0.1, p = 0.3), nor with maternal iron depletion (adjusted change in PWV in m/s = −0.2, 95% CI: −0.6, 0.2, p = 0.3). No evidence of association was observed between maternal sTfR or log R/F with infant bfPWV. However, anaemic mothers in the first half of pregnancy (Hb <11 g/dl) had infants with higher PWV by 1.0 m/s on average (95% CI: 0.1, 1.8, p = 0.02; table 3).

No association was observed between maternal intake of iron supplements at any stage in pregnancy and infant PWV (unadjusted change = −0.1, 95% CI: −0.5, 0.2). Adjusting for iron supplement intake did not alter the results of the models examining the association between maternal iron biomarkers with infant bfPWV, while it strengthened the association between maternal anaemia in early pregnancy and infant PWV (adjusted change = 1.2 m/s, 95% CI: 0.3, 2.1). Adjusting for infant heart rate attenuated the association between early pregnancy maternal anaemia and infant PWV (adjusted change = 0.4 m/s, 95% CI: −0.7, 1.4, p = 0.5).

## Discussion

PWV, a potential marker of cardiovascular health later in life, was ascertained in 284 babies aged 2–6 weeks at home in the Baby VIP study. We found no association between maternal iron status biomarkers in early pregnancy and infant PWV. However, maternal anaemia in the first half of pregnancy was associated with increased infant PWV.

The elastic properties of arteries vary along the arterial tree, with more elastic proximal arteries and stiffer distal ones. The amplitude of the pressure wave is higher in peripheral arteries than in central arteries. This ‘amplification phenomenon’ is known to be more pronounced in younger subjects [1]. In adults, average brachio-ankle PWV is approximately 20% higher than carotid-femoral PWV [20]. These reasons may explain the relatively higher bfPWV average in our study compared to the average newborn aortic PWV reported elsewhere [12, 21]. Another explanation could also be the way the distance was assessed between the two arterial sites (in a straight line). This is likely to be shorter than the distance the pulse wave travels along the vessel.

The ability of PWV measured very early in life to predict later cardiovascular health is unknown. Although there was no evidence of association with maternal iron status, this does not exclude the possibility that the latter may be linked to offspring cardiovascular indicators in adulthood. Therefore, long-term follow-up of a birth cohort with information on maternal iron status in pregnancy is required.

We found an association between early pregnancy maternal anaemia and infant arterial stiffness. Anaemia could reflect the extreme of the ID spectrum; however, only half of the anaemic women had sF <15 µg/l. Another study by the authors found no evidence of association between early pregnancy maternal Hb and offspring PWV at 10 years [22]. Maternal anaemia may have a true effect on infant arterial function, which may subside later in life. Alternatively, the observed association could be due to residual confounding by other causes of maternal poor health.

In a sensitivity analysis, we adjusted for infant heart rate in the association between maternal anaemia and infant PWV. This can improve the precision of the estimate if heart rate is strongly linked to the outcome. However, it cannot be considered a true confounder as it cannot affect maternal anaemia. Therefore, it was not included in the main model. Some studies have found heart rate to have a significant effect on arterial stiffness, while others have not [23, 24, 25, 26]. Infant heart rate could potentially be a mediator in the relationship between maternal anaemia and infant PWV if it is postulated that anaemic mothers are more likely to give birth to anaemic babies. However in this study, although infant heart rate was inversely associated with PWV, it was not associated with maternal anaemia (data not shown).

Our study assessed the exposure of interest prospectively, as the maternal serum samples were collected in the first trimester of pregnancy. We used the best available measure for iron status – the R/F ratio which relates directly to total body iron stores [15]. We assessed PWV, an innovative measure of cardiovascular health which causes less distress than measuring blood pressure in neonates/young infants, on a relatively large study population compared to other studies which have assessed PWV in this age group [12, 21]. Information on maternal Hb and iron supplements was ascertained objectively from the medical records, rather than by self-reporting.

A potential source of error in measuring PWV is the use of more distal conduit sites as a surrogate for less accessible central arteries [4]. However, the forearm circulation is where blood pressure is commonly measured, and lower limb arteries are commonly altered by atherosclerosis [1]. Estimating the actual distance between the recording sites using surface measurements could also be a source of error. The shorter the distance, the greater the absolute error in determining transit time [1]. However, our PWV data spread corresponds well with most other studies. Furthermore, any errors in measuring the distance between the arterial sites would be non-differential as the researcher was blind to the exposure. A further limitation is that we did not record infant blood pressure in this study.

This study demonstrates that infant arterial stiffness can be assessed using non-invasive techniques in population studies. Further research is needed to investigate the relationship between maternal nutrition during pregnancy and PWV in the child and adult offspring using a prospective study design to investigate the potential pathways underlying the developmental origins of cardiovascular disease.

## Figures and Tables

**Table 1:** Infant bfPWV in relation to measurement conditions and infant characteristics in the Baby VIP study

	Infant bfPWV, m/s			
	n	mean	SD	p
Sleeping status				<0.001^a^
Asleep	48	5.9	1.1	
Awake	239	6.8	1.3	
Position during measurement				<0.001^b^
In mother's arms	121	6.5	1.3	
Feeding in mother's arms	101	7.2	1.2	
In cot/on sofa or floor	61	6.1	1.1	
Measurement side				0.2^a^
Right	98	6.8	1.4	
Left	181	6.6	1.2	
Baby's age				0.7^a^
<28 days	206	6.6	1.3	
≥28 days	77	6.7	1.4	
Type of feeding				0.06^b^
Breast	122	6.9	1.4	
Bottled	109	6.5	1.3	
Mixed	53	6.4	1.2	

a Independent samples t test.

b One-way analysis of variance.

**Table 2:** Characteristics of participants in the Baby VIP study (n = 362) according to whether babies were followed up by home visits to measure PWV after hospital recruitment

	With PWV measurements			Without PWV measurements			p^a^
	total n	mean ± SD/n (%)/median	95% CI/IQR	total n	mean ± SD/n (%)/median	95% CI/IQR	
Gestational age, days	284	277 ± 14		78	276.3 ± 14		0.5
Birth weight, g	284	3,339 ± 636		78	3,293 ± 621		0.6
Maternal age at antenatal booking, years	284	31.1 ± 5.5		78	28.6 ± 6.0		0.0007
Maternal BMI at antenatal booking	281	26.5 ± 6.1		77	25.3 ± 4.9		0.1
IMD	284	28.6 ± 19.1		78	32.7 ± 19.6		0.1
Maternal Hb at ≤20 weeks’ gestation, g/dl	263	12.6 ± 1.0		66	12.4 ± 1.2		0.1
Maternal Hb at >20 weeks’ gestation, g/dl	266	11.6 ± 1.0		71	11.4 ± 1.1		0.2
First-trimester maternal sF, µg/l	273	33.4	17.4–61.6	75	28.6	13.1–68.7	0.5
First-trimester maternal sTfR, nmol/l	273	13.1	10.4–16.1	75	12.3	10.1–16.1	0.6
First-trimester maternal R/F ratio, µg/l	273	27.5	15.3–61.9	75	32.0	14.0–72.9	0.6
Primiparous	284	144 (51)	45, 57	78	29 (37)	27, 49	0.03
Male baby	284	137 (48)	42, 54	78	45 (58)	47, 69	0.1
Maternal White ethnicity	284	220 (78)	72, 82	78	68 (87)	78, 94	0.1
Maternal smoking at antenatal booking	278	36 (13)	9, 18	76	13 (17)	9, 28	0.5
Gestational diabetes	284	5 (2)	1, 4	78	1 (1)	0, 7	0.1
Pre-eclampsia	284	3 (1.1)	0,3.1	78	3 (3.9)	1.0, 10.8	0.1
Anaemia at ≤20 weeks’ gestation (<11 g/dl)	263	10 (4)	2, 7	66	6 (9)	3, 19	0.1
Anaemia at >20 weeks’ gestation (<10.5 g/dl)	266	35 (13.2)	9.3, 1.8	71	13 (18.3)	10.1, 29.3	0.3
Had taken iron supplements in pregnancy	284	92 (32)	27, 38	77	29 (38)	27, 49	0.4
Had taken multivitamin supplements in pregnancy	284	157 (55)	49, 61	77	32 (42)	30, 53	0.03

a Independent samples t test or Mann-Whitney test for continuous variables, and χ^2^ test for categorical variables.

**Table 3:** Associations of infant bfPWV at 2–6 weeks with indicators of iron status during pregnancy in the Baby VIP study

Predictor	Change in infant bfPWV (m/s)						
	unadjusted	95% CI	p	adjusted^a^	adjusted 95% CI	p	n (multi-variable model)
Maternal sF at 12 weeks’ gestation (per 10-µg/l change)	0.02	–0.01, 0.1	0.2	0.02	–0.01, 0.1	0.3	261
Maternal iron depletion at 12 weeks’ gestation (sF <15 µg/l)	–0.2	–0.5, 0.3	0.4	–0.2	–0.6, 0.2	0.3	261
Maternal sTfR at 12 weeks’ gestation (nmol/l)	0.03	–0.004, 0.1	0.1	0	–0.01, 0.04	0.3	261
Maternal log R/F ratio at 12 weeks’ gestation (µg/l)	0	–0.1, 0.1	0.9	0	–0.2, 0.1	0.5	261
Maternal Hb at ≥20 weeks’ gestation (g/dl)	0.1	–0.1, 0.3	0.3	0.1	–0.1, 0.2	0.6	253
Maternal Hb at >20 weeks’ gestation (g/dl)	0.2	–0.001, 0.3	0.05	0.2	–0.004, 0.3	0.06	256
Maternal anaemia at ≥20 weeks’ gestation (<11 g/dl)	0.7	–0.1, 1.6	0.08	1.0	0.1, 1.9	0.02	253
Maternal anaemia at >20 weeks’ gestation (<10.5 g/dl)	–0.1	–0.5, 0.4	0.8	0.01	–0.5, 0.5	0.9	256

a Adjusted for baby's age, PWV measurement circumstances (position, feeding, asleep or awake, side), maternal age, smoking, gestational diabetes, preeclampsia, blood pressure at booking and 36 weeks’ gestation, deprivation score, and customised birth weight centile (takes into account maternal pre-pregnancy weight, height, ethnicity, parity, gestational age and baby's sex).
